# Supplementary figures and images for: The triad interaction of ULK1, ATG13, and FIP200 is required for ULK complex formation and autophagy
Source: eLife. 2025 Jun 24;13:RP101531. doi: 10.7554/eLife.101531 (PMC12187132; doi:10.7554/eLife.101531)

Figure 1E FIP200

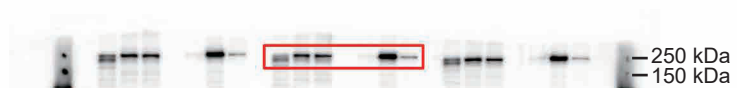

Figure 1E FLAG

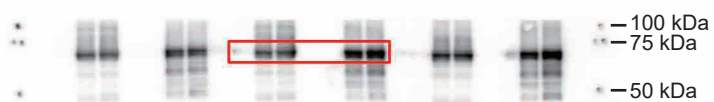

Figure 1E ULK1

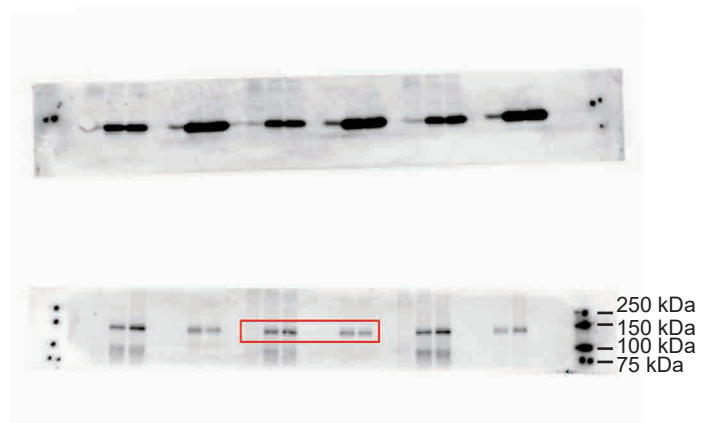

Supplement: Figure 1—source data 1. [file elife-101531-fig1-data1.pdf]

Figure 1-figure supplement 2

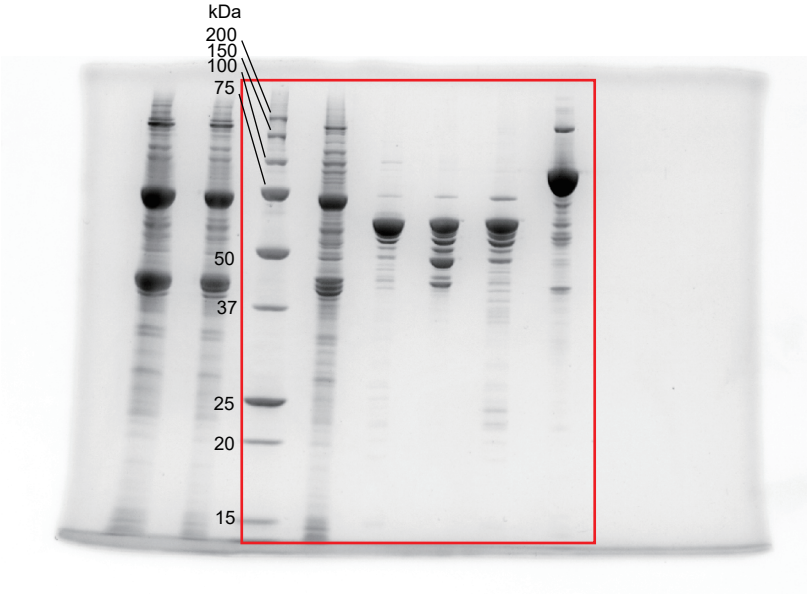

Supplement: Figure 1—figure supplement 2—source data 1. [file elife-101531-fig1-figsupp2-data1.pdf]

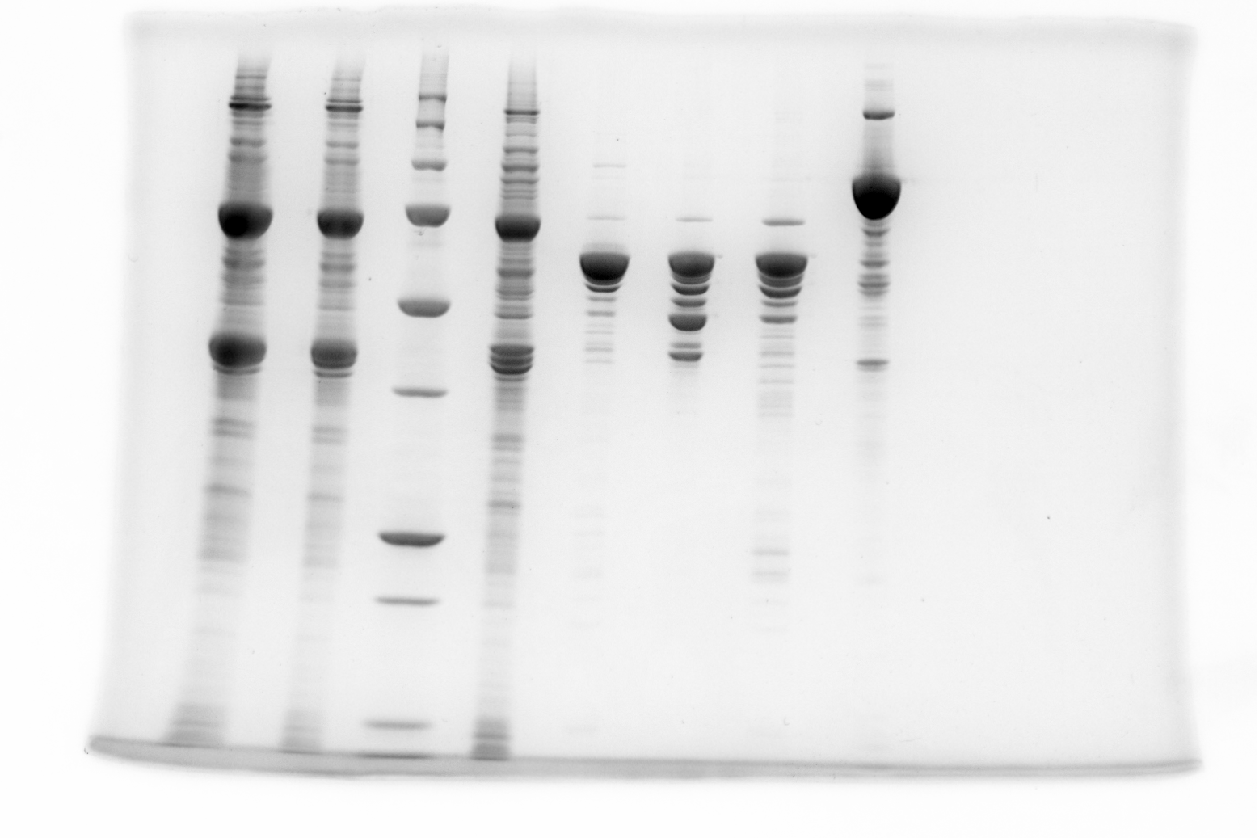

Supplement: Figure 1—figure supplement 2—source data 2. [file elife-101531-fig1-figsupp2-data2.zip › Figure1-figure spplement2-source data2.tif]

Figure 2D ATG13-FLAG

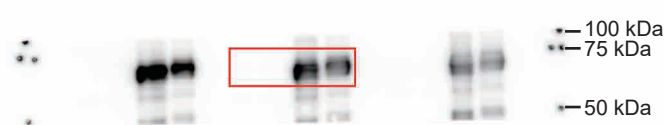

Figure 2D b-actin

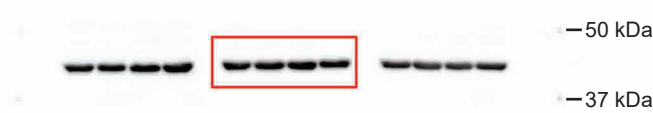

Figure 2D FIP200

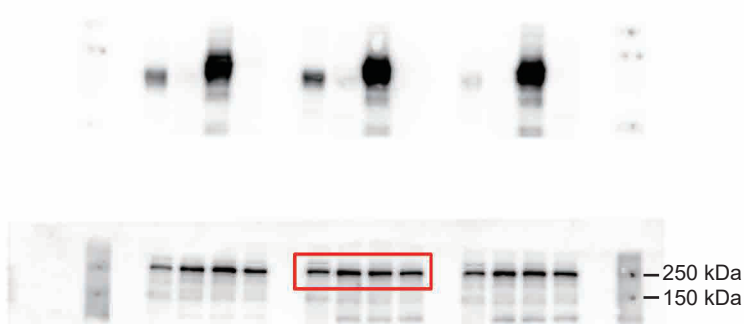

Figure 2D ULK1

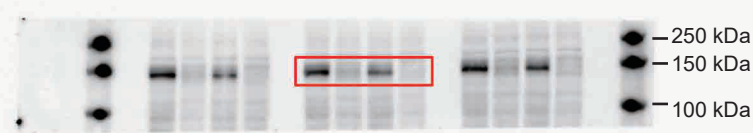

Supplement: Figure 2—source data 1. [file elife-101531-fig2-data1.pdf]

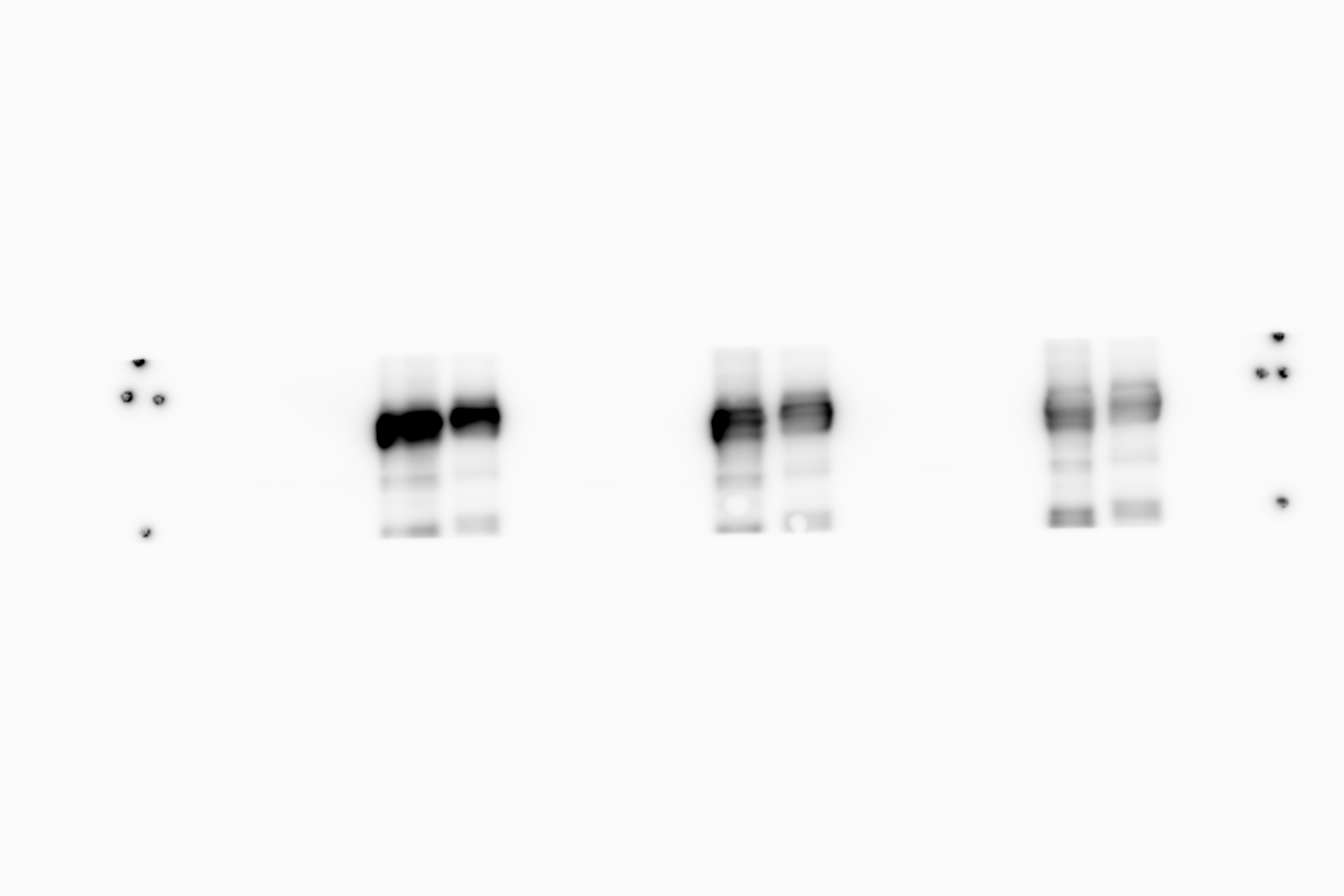

Supplement: Figure 2—source data 2. [file elife-101531-fig2-data2.zip › Figure2D_ATG13-FLAG_raw.tif]

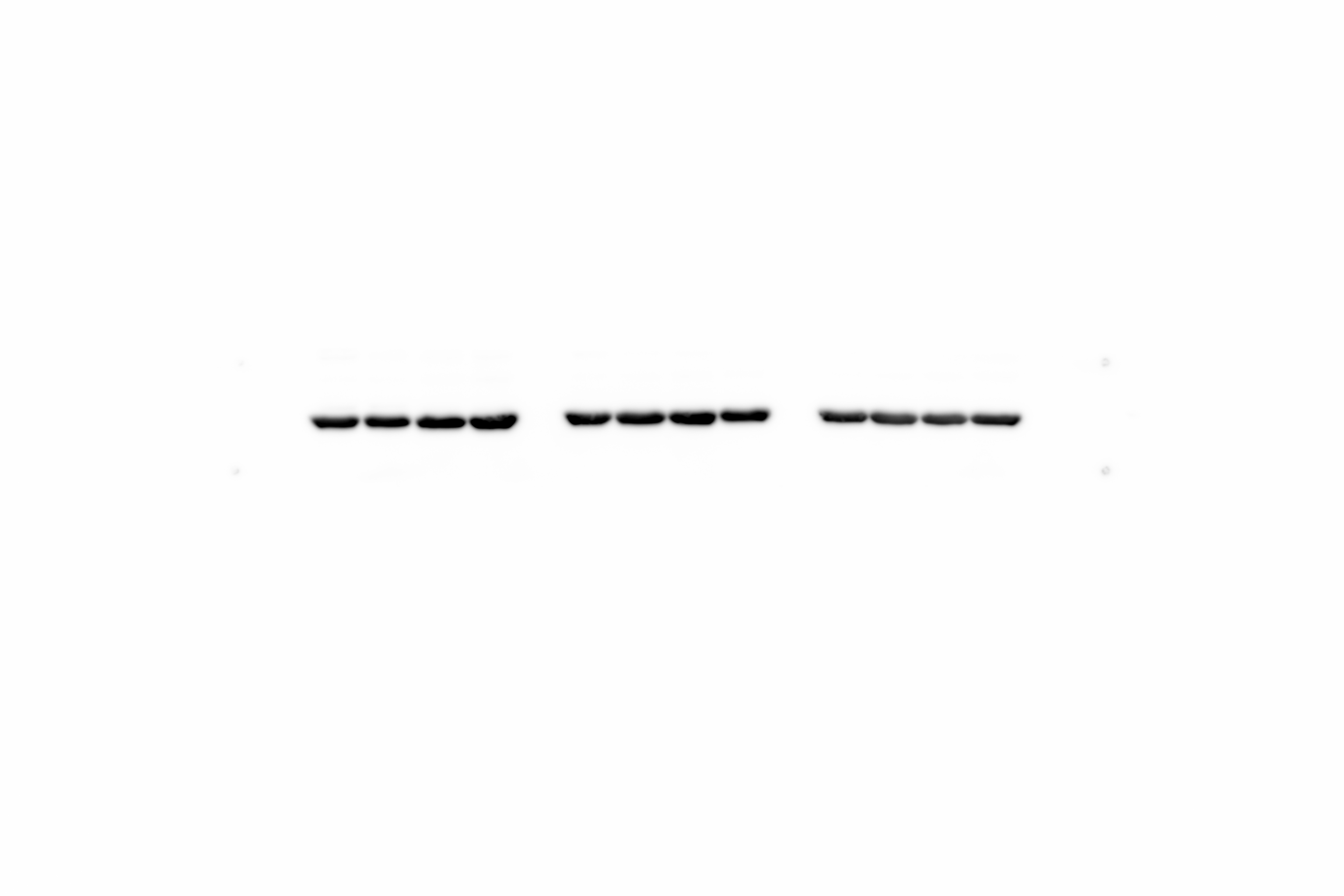

Supplement: Figure 2—source data 2. [file elife-101531-fig2-data2.zip › Figure2D_b-actin_raw.tif]

Figure 3C

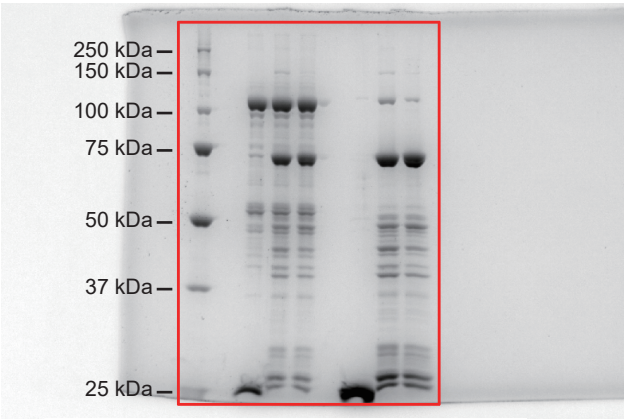

Figure 3E ATG13

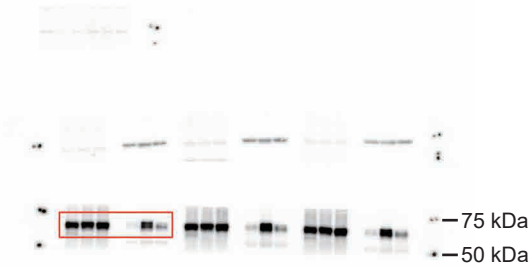

Figure 3E FIP200

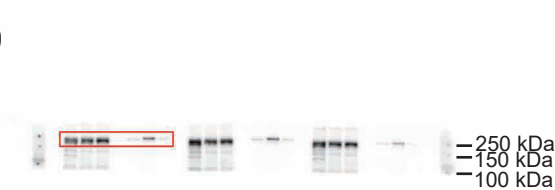

Figure 3E FLAG-ULK1

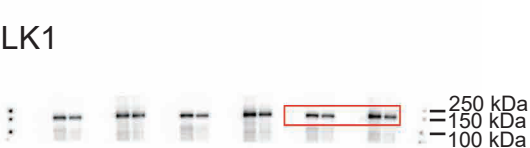

Figure 3G

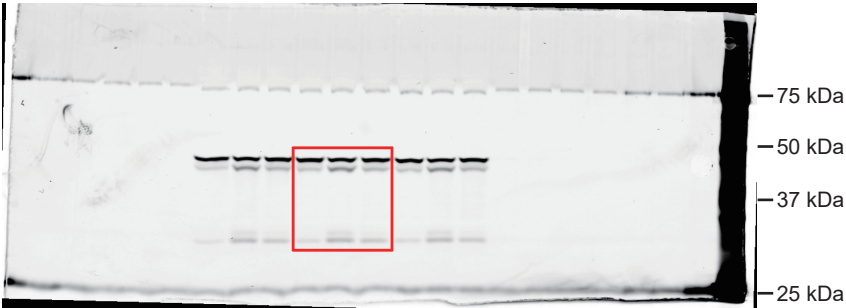

Supplement: Figure 3—source data 1. [file elife-101531-fig3-data1.pdf]

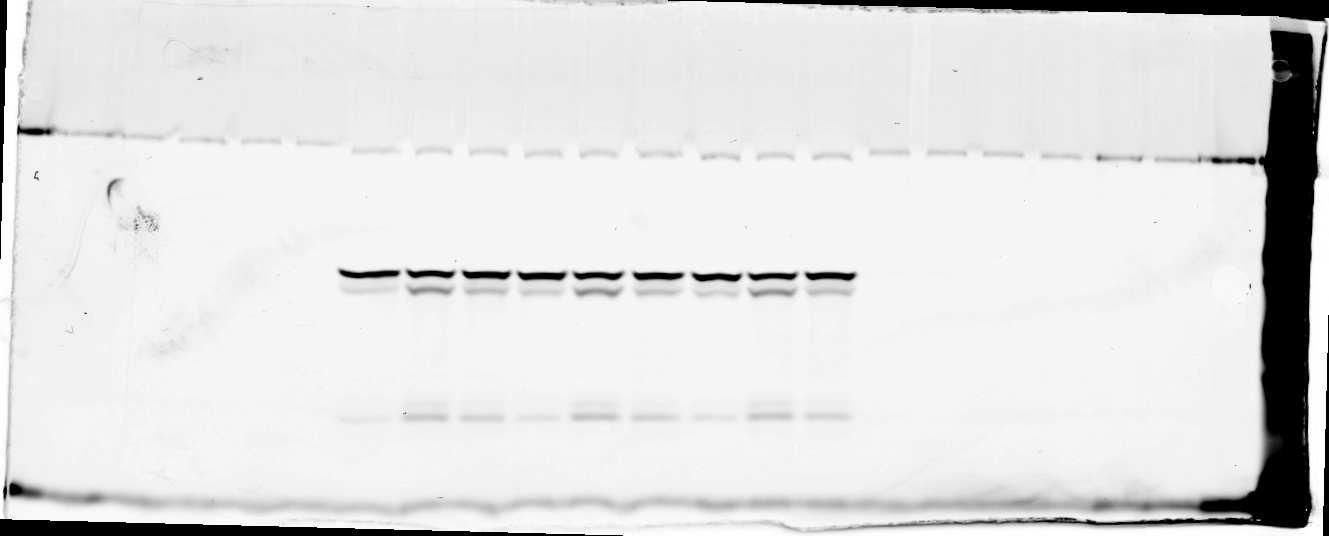

Supplement: Figure 3—source data 2. [file elife-101531-fig3-data2.zip › Figure3G_raw.tif]

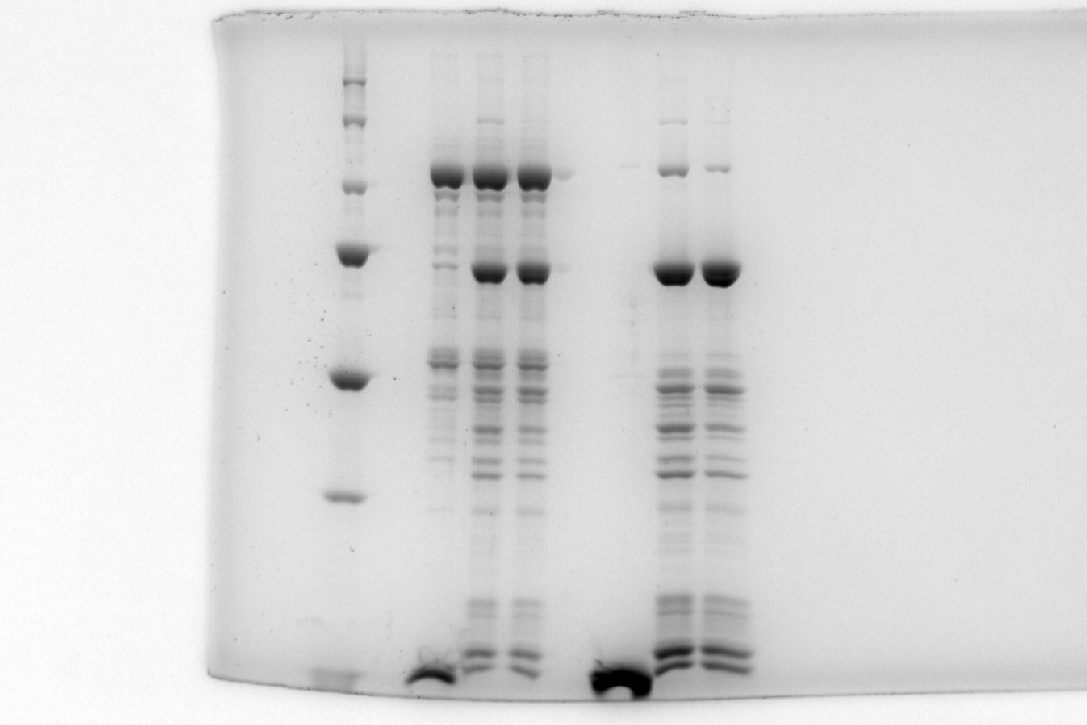

Supplement: Figure 3—source data 2. [file elife-101531-fig3-data2.zip › Figure3C_raw.tif]

Figure 4B ATG13-FLAG

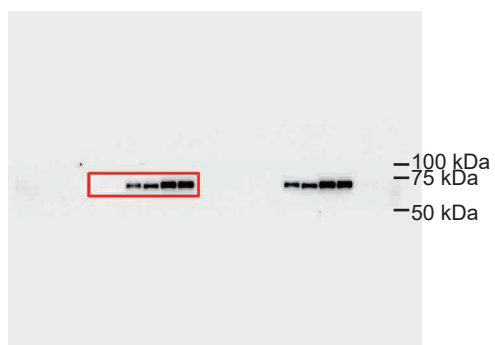

Figure 4B b-actin

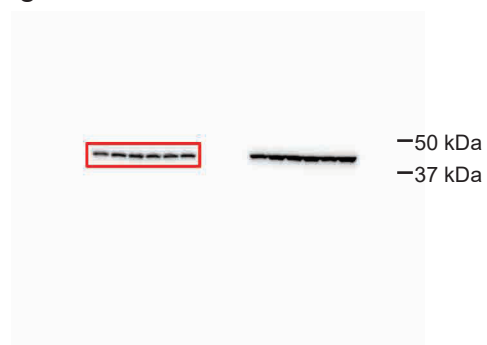

Figure 4B FIP200

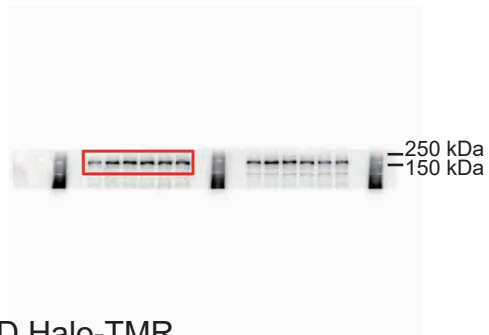

Figure 4B ULK1

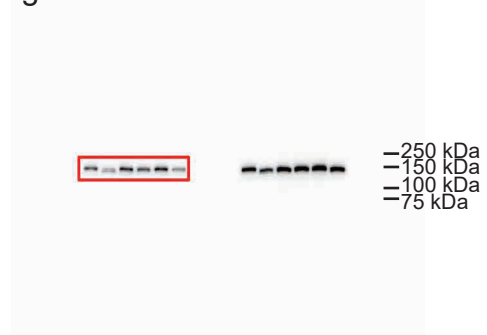

Figure 4D Halo-TMR

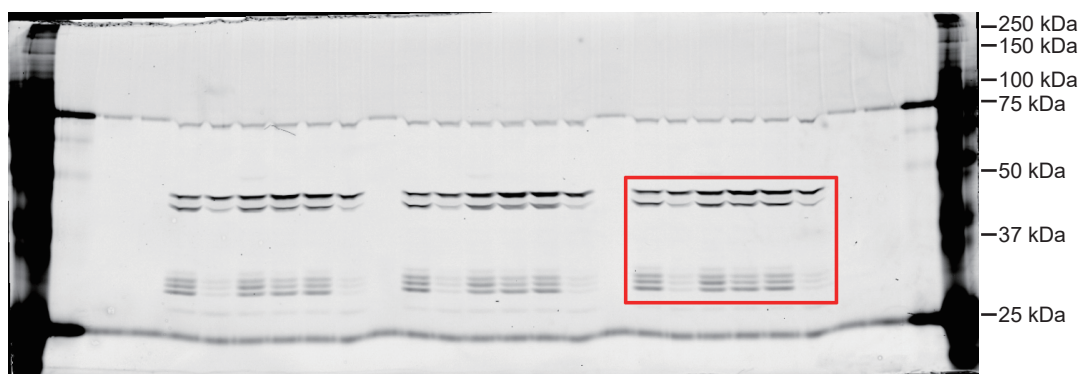

Supplement: Figure 4—source data 1. [file elife-101531-fig4-data1.pdf]

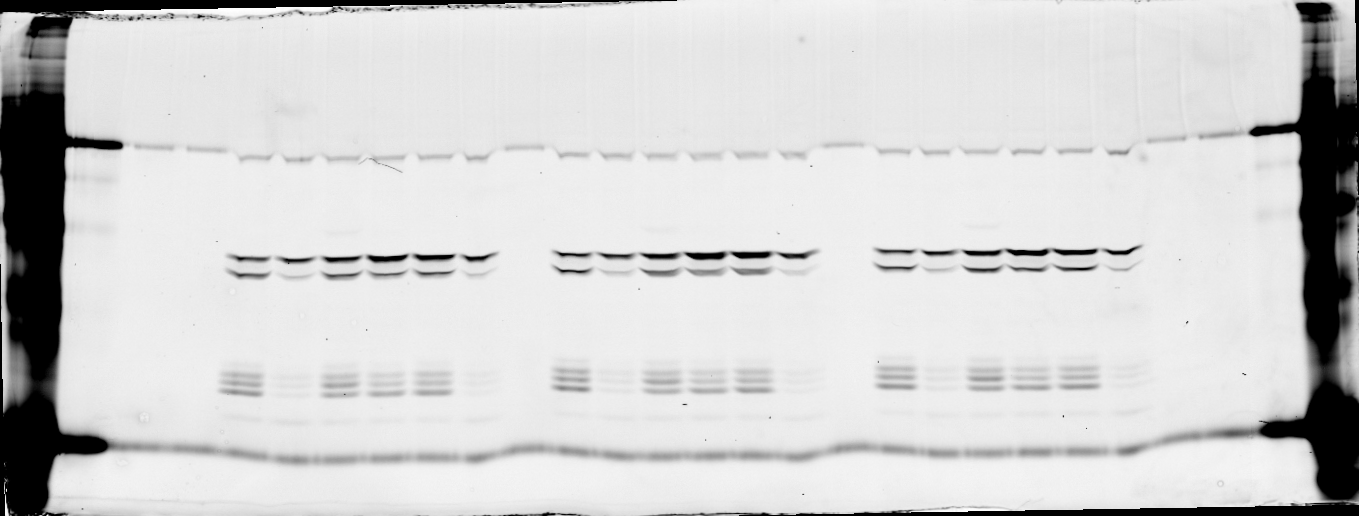

Supplement: Figure 4—source data 2. [file elife-101531-fig4-data2.zip › Figure4D_Halo-TMR(In-gel fluo)_raw.tif]

Figure 4-figure supplement 1A

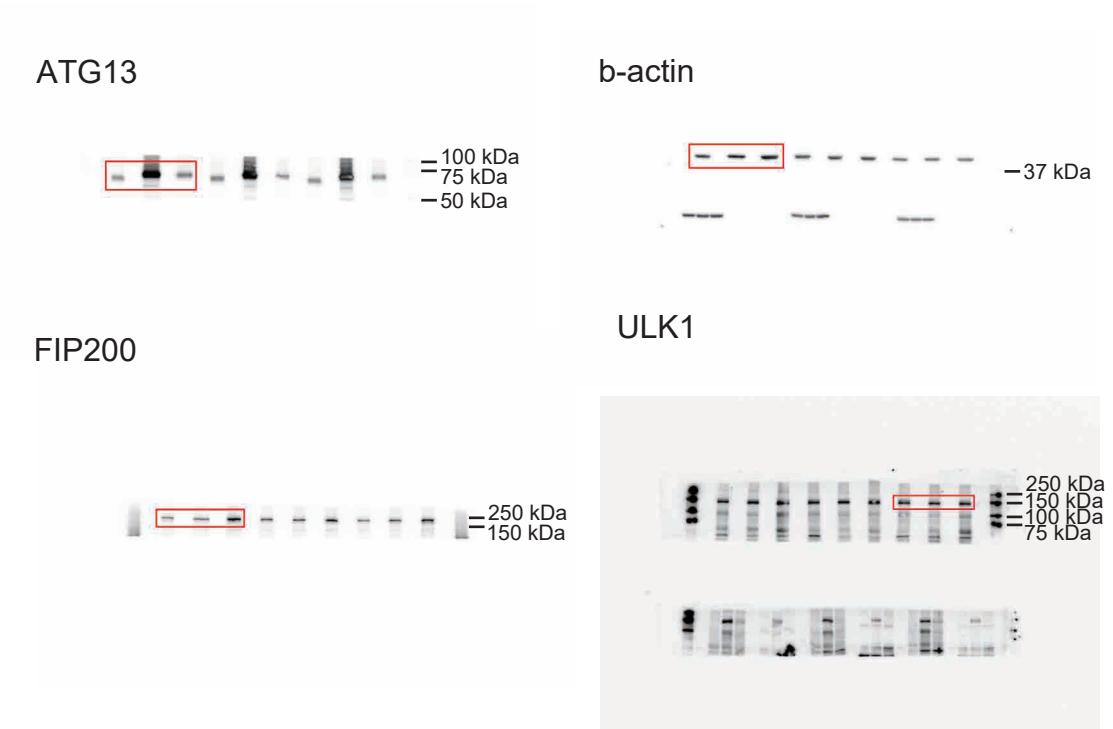

Figure 4-figure supplement 1D

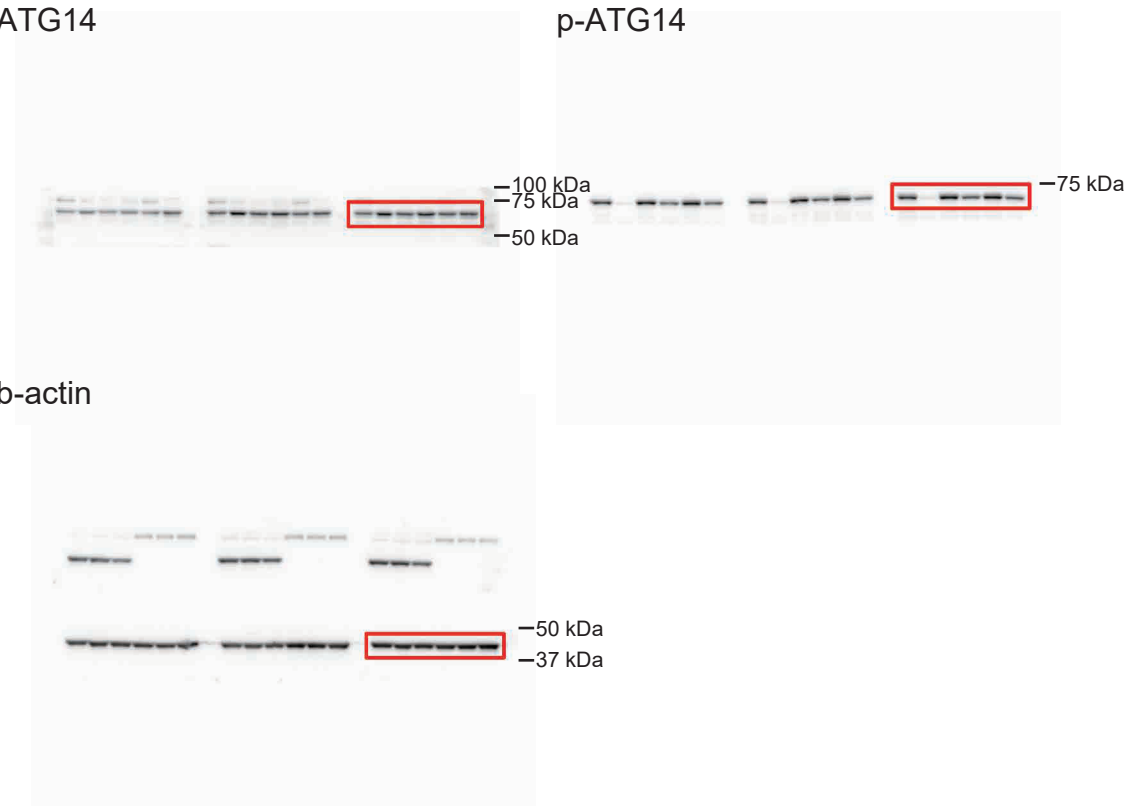

Supplement: Figure 4—figure supplement 1—source data 1. [file elife-101531-fig4-figsupp1-data1.pdf]
